# Gastric and intestinal phenotypic marker expression in gastric carcinomas and recurrence pattern after surgery-immunohistochemical analysis of 213 lesions-

**DOI:** 10.1038/sj.bjc.6602147

**Published:** 2004-08-31

**Authors:** Y Tajima, K Yamazaki, N Nishino, K Morohara, T Yamazaki, T Kaetsu, S Suzuki, M Kawamura, K Kumagai, M Kusano

**Affiliations:** 1 Department of Surgery, Division of General & Gastroenterological Surgery, Showa University, School of Medicine, 1-5-8, Hatanodai, Shinagawa-ku, Tokyo 142-8666, Japan; 2 Department of Surgery, Toyosu Hospital, Showa University, 1-18, Toyosu 4 chome, Koto-ku, Tokyo 135-8577, Japan

**Keywords:** gastric carcinoma, immunohistochemistry, human gastric mucin, MUC6, MUC2, CD10, recurrence pattern

## Abstract

Both gastric and intestinal phenotypic markers are known to be expressed in gastric carcinomas, irrespective of their histologic type. In the present study, the relation between gastric and intestinal phenotypic marker expression in gastric carcinomas and the recurrence pattern after surgery was examined. The phenotypic marker expression of the tumour was determined by examining the expression of human gastric mucin (HGM), MUC6, MUC2 and CD10 in 213 advanced gastric carcinomas in 213 patients who had undergone a curative resection (97 died from recurrent gastric carcinoma and 116 were alive without recurrence at the end of the follow-up period). Tumours were classified into gastric (G), gastric and intestinal mixed (GI), intestinal (I) or unclassified (UC) phenotypes according to the immunopositivity of HGM, MUC6, MUC2 and CD10 stainings. The incidence of HGM-positive tumours and MUC2-negative tumours was significantly higher in tumours with peritoneal recurrence than in tumours without recurrence (73.3%, 44 out of 60 cases *vs* 54.3%, 63 out of 116 (*P*=0.022); and 70.0%, 42 out of 60 *vs* 38.8%, 45 out of 116 (*P*=0.0002), respectively). The incidence of G-phenotype tumours was also significantly higher in tumours with peritoneal recurrence than in tumours without recurrence (58.3%, 35 out of 60 cases *vs* 28.4%, 33 out of 116 (*P*=0.0002)). The incidence of MUC2-negative tumours and CD10-positive tumours was significantly higher in tumours with haematogenous recurrence than in tumours without recurrence (62.5%, 20 out of 32 cases *vs* 38.8%, 45 out of 116 (*P*=0.028); and 43.8%, 14 out of 32 *vs* 23.3%, 27 out of 116 (*P*=0.039); respectively). Our present findings show that the gastric and intestinal phenotypic marker expression of the tumour, determined by immunohistochemical staining for HGM, MUC6, MUC2 and CD10, can be used to predict the pattern of gastric carcinoma recurrence after curative resection.

Gastric carcinoma is histologically classified in two manners, differentiated *vs* undifferentiated, or intestinal *vs* diffuse, based on the tendency of gland formation (Lauren, 1965; Nakamura *et al*, 1968). However, recent mucin histochemical and immunohistochemical examinations have demonstrated that gastric and intestinal phenotypic cell markers are widely expressed in gastric carcinomas, irrespective of their histologic type (Tatematsu *et al*, 1990; Egashira *et al*, 1999; Endoh *et al*, 1999, 2000; Koseki *et al*, 2000; Saito *et al*, 2001; Tajima *et al*, 2001a, 2001b, 2003; Tsukashita *et al*, 2001; Kabashima *et al*, 2002; Shibata *et al*, 2003). Several authors have reported that gastric carcinomas can be classified as having either a gastric (G), gastric and intestinal mixed (GI) or intestinal (I) phenotype, depending on the immunopositivity of human gastric mucin (HGM), MUC6, MUC2 and CD10 stainings (Tajima *et al*, 2001b, 2003; Kabashima *et al*, 2002). Human gastric mucin, MUC6, MUC2 and CD10 specifically express in gastric surface mucous cells, pyloric gland cells, intestinal goblet cells of the mature gastrointestinal tract and brush border of intestinal epithelial cells, respectively. We previously reported that G-phenotype tumours accounted for 27.7% of differentiated tumours, often referred to as intestinal-type tumours according to Lauren (1965), while I-phenotype tumours accounted for 10.1% of undifferentiated tumours (Tajima *et al*, 2001b). The phenotypic marker expression of tumours is conventionally thought to imitate that of the tissue of origin. Thus, the previous data described above suggest that gastric carcinomas can occur in various types of gastric mucosa, although differentiated tumours have generally been considered to arise from gastric mucosa with intestinal metaplasia and undifferentiated tumours to arise from ordinary gastric mucosa without intestinal metaplasia (Lauren, 1965; Nakamura *et al*, 1968). Recently, G-phenotype tumours have been associated with poorer outcome and greater malignant potential in the incipient phase of invasion and metastasis, compared with those of other phenotype tumours (Endoh *et al*, 1999; Koseki *et al*, 2000; Saito *et al*, 2001; Tajima *et al*, 2001b; Kabashima *et al*, 2002; Shibata *et al*, 2003). Therefore, phenotypic classification could be useful not only for investigating the tumorigenesis of gastric carcinoma, but also for evaluating tumour aggressiveness. Phenotypic expression in gastric carcinomas has also been reported to be strongly dependent on genetic changes (Endoh *et al*, 2000).

Early tumour detection, standardised surgical treatment with systematic lymph node dissection and appropriate chemotherapy have improved the survival of patients with gastric carcinoma (Otsuji *et al*, 1998). However, recurrences are likely to assume a variety of forms in various different organs, even after a curative resection of the gastric carcinoma. Risk factors for peritoneal recurrence have been reported to include an undifferentiated histological type, infiltrative tumour growth and serosal invasion, while risk factors for haematogenous recurrence have been reported to include a vessel invasion, differentiated histological type and expansive tumour growth (Maehara *et al*, 1996; Yoo *et al*, 2000; Ohno *et al*, 2003; Roviello *et al*, 2003). The prediction of recurrence patterns after surgery could help better follow-up programmes and appropriate treatment strategies to be designed for gastric carcinoma patients. However, the relation between gastric and intestinal phenotypic marker expression in gastric carcinomas and the recurrence pattern after surgery has not been investigated.

In the present study, we examined surgically resected tumour specimens from 213 patients with advanced gastric carcinoma using HGM, MUC6, MUC2 and CD10 immunohistochemical stainings to clarify the relation between gastric and intestinal phenotypic marker expression in gastric carcinomas and the recurrence pattern after surgery.

## MATERIALS AND METHODS

### Patients

**Figure 1:**
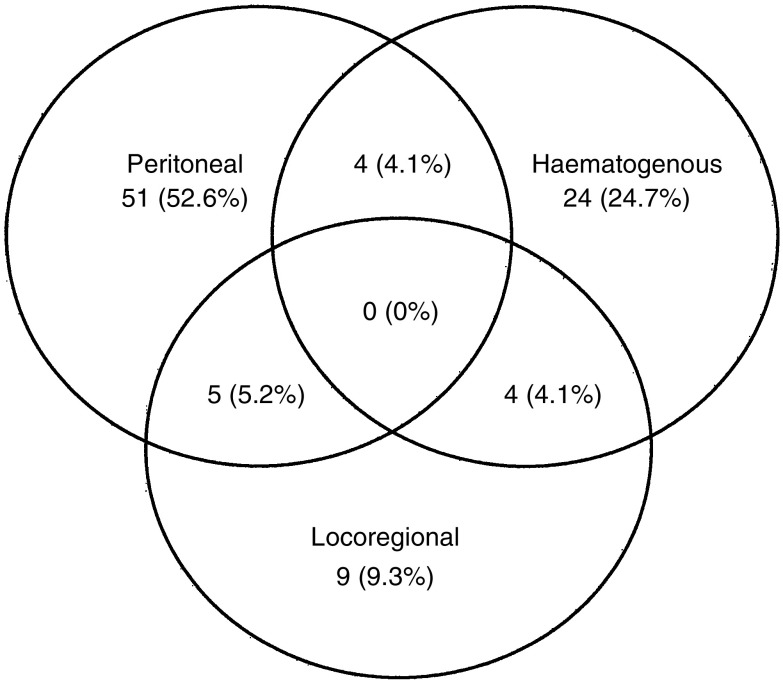
Recurrence patterns in 97 patients after curative resection.

Our series consisted of 213 patients who had undergone potentially curative resections for advanced gastric carcinoma between 1994 and 1998 at the Showa University Hospital and the Toyosu Hospital, Showa University, Tokyo, Japan. A total gastrectomy, or a distal or proximal subtotal gastrectomy with lymph node dissection, was performed, depending on the tumour location and surgical margin. Each patient underwent D2 (141 cases) or D3 (72 cases) lymphadenectomy and a mean of 28.3±11.8 lymph nodes per patient was dissected. As for the chemo or radiation therapies after surgery, all patients received oral administration of 5-fluorouracil (5-FU; 200 mg body^−1^ day^−1^) for more than 2 months after the operation. None of the patients received radiotherapy after surgery. After operation, routine follow-up consisted of a physical examination, blood examination (including carcinoembryonic antigen, CA19-9 levels), radiological examination, endoscopic examination, computed tomography and ultrasonography. Follow-up examinations were performed every 3 months for the first postoperative year and then at intervals of between 6 and 12 months for at least 5 years. Of the 213 patients in our series, 116 patients were still alive with no recurrences at the time of the last follow-up (control group) and 97 had died from recurrent gastric carcinoma. The recurrence sites in the 97 patients were precisely determined by radiological examination, endoscopic examination, computed tomography, ultrasonography or scintigraphy, and the recurrence pattern was divided into three groups: peritoneal, haematogenous and locoregional recurrences. Peritoneal recurrence occurred in 60 patients, haematogenous recurrence occurred in 32 patients and locoregional recurrence occurred in 18 patients (Figure 1

). Haematogenous recurrence occurred in the liver in 29 patients, in the lung in five patients and in the brain in three patients. Locoregional recurrence occurred in the lymph node, in 16 patients and in the anastomosis or stump in two patients.

### Clinicopathologic review

Serial 5-mm-thick tissue sections of the entire tumour were prepared from the resected specimens fixed with 10% buffered formalin, embedded in paraffin and stained with haematoxylin and eosin (H&E). Clinicopathologic findings such as age, gender, tumour size, tumour location, depth of invasion, growth pattern, lymphatic invasion, blood vessel invasion and lymph node metastasis were reviewed according to the Japanese Classification of Gastric Carcinoma (Japanese Gastric Cancer Association, 1998). In the present study, the tumours were classified histologically as either differentiated or undifferentiated, according to the criteria described by Nakamura *et al* (1968).

### Immunohistochemical staining

The following mouse monoclonal antibodies were used: 45M1 (Novocastra Laboratories Ltd, UK), diluted 1 : 50, to detect HGM; CLH5 (Novocastra Laboratories Ltd), diluted 1 : 50, to detect MUC6 glycoprotein; Ccp58 (Novocastra Laboratories Ltd), diluted 1 : 100, to detect MUC2 glycoprotein; and 56C6 (Novocastra Laboratories Ltd), diluted 1 : 40, to detect CD10 glycoprotein expression. 45M1 and CLH5 were examined as G-phenotype markers, and Ccp58 and 56C6 were examined as I-phenotype markers. 45M1 recognises the mucin epitope located in the peptide core of HGM, which is synonymous with MUC5AC. This antibody is known to react with surface foveolar cells in the stomach (Bara *et al*, 1998; Nollet *et al*, 2002). MUC6 glycoprotein is expressed in mucous cells of the neck zone of oxyntic mucosa and in antral glands (De Bolos *et al*, 1995; Reis *et al*, 1999; Machado *et al*, 2000). MUC2 glycoprotein, also known as the ‘intestinal-mucin-related protein antigen’, is an intestinal apomucin and is known to be also expressed in the supranuclear area of the goblet cells in regions showing intestinal metaplasia in the stomach (Kim and Gum, 1995; Sakamoto *et al*, 1997; Baldus *et al*, 1998; Utsunomiya *et al*, 1998; Reis *et al*, 1999; Machado *et al*, 2000). CD10 glycoprotein is a 100-kDa cell metalloendopeptidase that inactivates a variety of biologically active peptides and is known to be expressed on the brush border of intestinal epithelial cells as well as in the germinal centres of lymphoid follicles and the microvilli of the kidney (Ronco *et al*, 1984; Trejdosiewicz *et al*, 1985).The avidin–biotinyl-peroxidase complex immunohistochemical method was used for all immunohistochemical studies according to a previously described protocol (Hsu *et al*, 1981).

**Figure 2:**
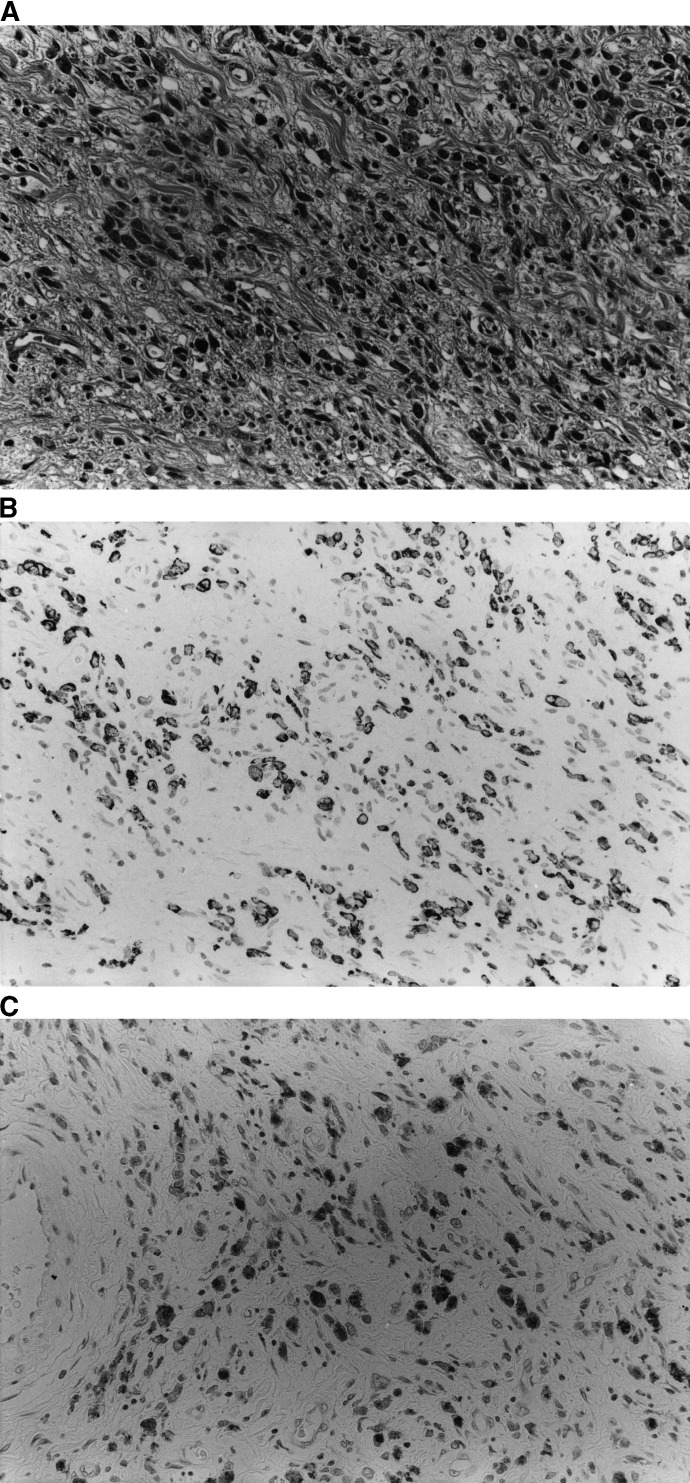
(**A–C**) Gastric carcinoma of the G-phenotype. (**A**) Histological features (H&E, original magnification × 400). (**B**) Human gastric mucin is expressed in the cancer cell cytoplasm (45M1, original magnification × 400). (**C**) MUC6 glycoprotein is also expressed in the cancer cell cytoplasm (CLH5, original magnification × 400).

**Figure 3:**
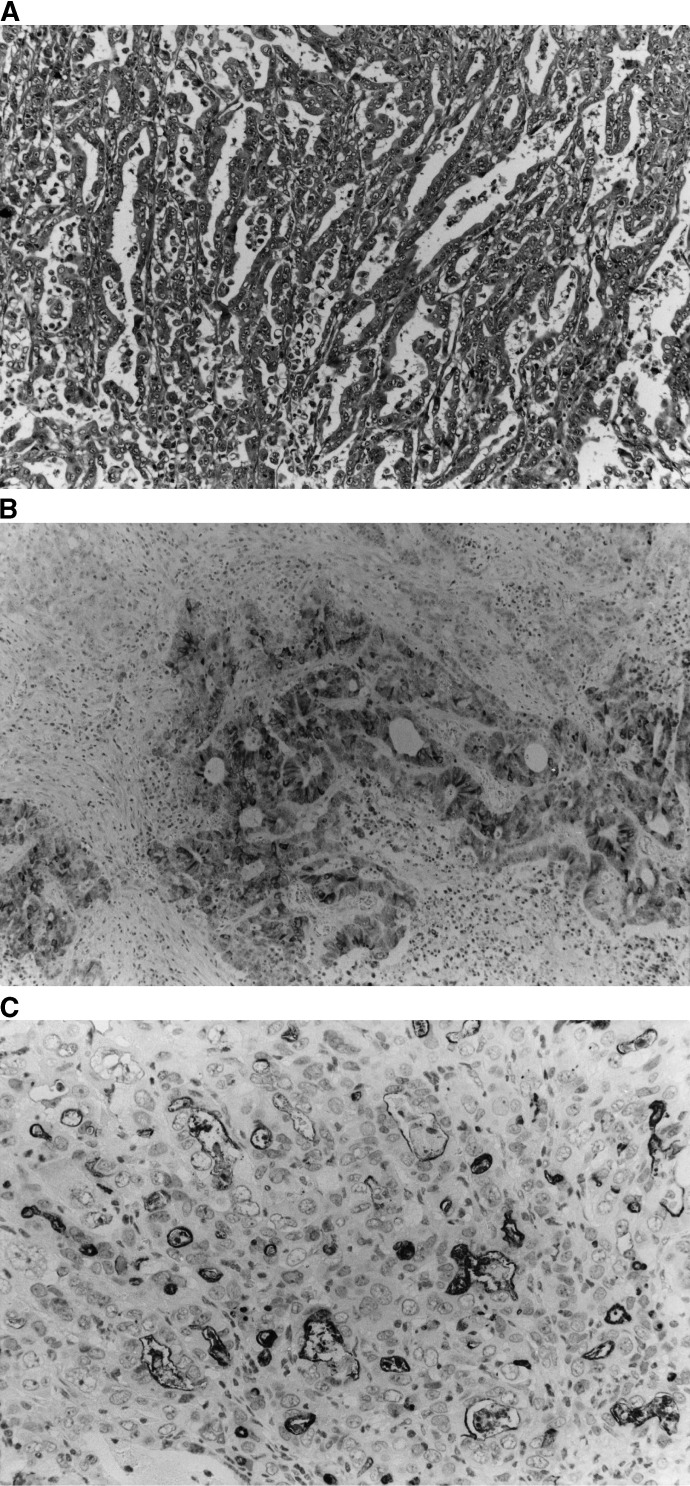
(**A–C**) Gastric carcinoma of the I-phenotype. Histological features (H&E, original magnification × 200). (**B**) MUC2 glycoprotein is expressed in the cancer cell cytoplasm (Ccp58, original magnification × 200). (**C**) CD10 glycoprotein is also expressed on the luminal surfaces of cancerous glands (56C6, original magnification × 200).

With regard to the evaluations of HGM, MUC6, MUC2 and CD10 staining, distinct staining in more than 5% of the tumour cells was recorded as positive immunoreactivity for the relevant marker. These immunohistochemical methods were used to classify the tumours into four different phenotypes: tumours with G-phenotypic cells accounting for more than 5% of their cell population were classified as G- phenotype carcinomas (Figure 2

); those with I-phenotypic cells accounting for more than 5% of their cell population were classified as I-phenotype carcinomas (Figure 3

); those with both gastric and intestinal phenotypic cells accounting for more than 5% of their cell population were classified as GI-phenotype carcinomas; and those with both gastric and intestinal phenotypic cells accounting for less than 5% of their cell population were regarded as carcinomas of UC-phenotype (Tajima *et al*, 2001b, 2003; Tsukashita *et al*, 2001; Yao *et al*, 2002).

The histopathological and immunohistochemical examinations were independently performed by two observers (Tajima and Yamazaki). The results were then compared, and any discrepancies were resolved by consensus after further histopathological review.

### Statistical analysis

Statistical calculations were performed using the Stat View software package (version 5.0; Abacus Concepts, Inc., Berkeley, CA, USA). The data were analysed using Student' *t*-test or Mann–Whitney *U*-test, in addition to the *χ*^2^ test or Fisher' exact test. Differences of *P*<0.05 were considered significant.

## RESULTS

### Relations between recurrence patterns and clinicopathological features

The relations between the recurrence patterns and the clinicopathological features are shown in Table 1

**Table 1:** Relations between recurrence patterns and clinicopathological characteristics

	**Peritoneal (*n*=60)**	**Haematogenous (*n*=32)**	**Locoregional (*n*=18)**	**Control (*n*=116)**
*Age (years)*^a^	64.1±14.5	67.9±14.0	69.0±12.3	63.1±11.6
				
*Gender*^b^				
Male	33 (55.0%)	27 (84.4%)	16 (88.9%)	71 (61.2%)
Female	27 (45.0%)	5 (15.6%)	2 (11.1%)	45 (38.8%)
				
*Tumour size*^c^				
60 mm⩾	15 (25.0%)	15 (46.9%)	7 (38.9%)	69 (59.5%)
60 mm<	45 (75.0%)	17 (53.1%)	11 (61.1%)	47 (40.5%)
				
*Tumour location*^d^				
Upper	15 (25.0%)	12 (37.5%)	5 (27.8%)	34 (29.3%)
Middle	16 (26.7%)	6 (18.8%)	5 (27.8%)	39 (33.6%)
Lower	19 (31.7%)	10 (31.3%)	7 (38.9%)	40 (34.5%)
Whole	10 (16.7%)	4 (12.5%)	1 (5.6%)	3 (2.6%)
				
*Histologic type*^e^				
Differentiated	17 (28.3%)	16 (50.0%)	8 (44.4%)	61 (52.6%)
Undifferentiated	43 (71.7%)	16 (50.0%)	10 (55.6%)	55 (47.4%)
				
*Depth of invasion*^f^				
mp. ss	8 (13.3%)	15 (46.9%)	6 (333%)	63 (54.3%)
se, si	52 (86.7%)	17 (53.1%)	12 (66.7%)	53 (45.7%)
				
*Growth pattern*^g^				
Expansive	18 (30.0%)	18 (56.3%)	8 (44.4%)	67 (57.8%)
Infiltrative	42 (70.0%)	14 (43.8%)	10 (55.6%)	49 (42.2%)
				
*Lymphatic invasion*				
Negative	7 (11.7%)	4 (12.5%)	2 (11.1%)	24 (20.7%)
Positive	53 (88.3%)	28 (87.5%)	16 (88.9%)	92 (79.3%)
				
*Blood vessel invasion*^h^				
Negative	37 (61.7%)	11 (34.4%)	6 (33.3%)	67 (57.8%)
Positive	23 (38.3%)	21 (65.6%)	12 (66.7%)	49 (42.2%)
				
*Lymph node metastasis*^i^				
Negative	7 (11.7%)	4 (12.5%)	4 (22.2%)	47 (40.5%)
Positive	53 (883%)	28 (87.5%)	14 (77.8%)	69 (59.5%)

mp=muscularic propria; ss=subserasa; se=serosa exposed; si=serosa-infiltrating adjacent tissue.

a *P*=0.016 (haematogenous *vs* control).

b *P*=0.0057 (peritoneal *vs* haematogenous) and P=0.02 (haematogenous *vs* control).

c *P*<0.001 (peritoneal *vs* control).

d *P*=0.012 (peritoneal *vs* control).

e *P*=0.0036 (peritoneal *vs* control).

f *P*=0.001 (peritoneal *vs* haematogenous) and *P*<0.0001 (peritoneal *vs* control).

g *P*=0.026 (peritoneal *vs* haematogenous) and *P*=0.0009 (peritoneal *vs* control).

h *P*=0.023 (peritoneal *vs* haematogenous) and *P*=0.032 (haematogenous *vs* control).

i *P*=0.0002 (peritoneal *vs* control) and *P*=0.0061 (haematogenous *vs* control).

. Peritoneal recurrence was significantly associated with a larger tumour size (*P*<0.0001), a deeper depth of invasion (*P*<0.0001) and higher incidences of tumours with whole tumour location (*P*=0.012), undifferentiated-type tumours (*P*=0.0036), infiltrative growth tumours (*P*=0.0009) and lymph node metastasis (*P*=0.0002), compared with the control group. Haematogenous recurrence was significantly associated with a higher mean age (*P*=0.016), a higher male-to-female ratio (*P*=0.02) and higher incidences of blood vessel invasion (*P*=0.032) and lymph node metastasis (*P*=0.0061), compared with the control group. No significant differences in the evaluated clinicopathological factors were observed between patients with locoregional recurrence and the control group.

### Relations between recurrence patterns and expressions of HGM, MUC6, MUC2 and CD10

The relations between the recurrence patterns and the expressions of HGM, MUC6, MUC2 and CD10 are shown in Table 2

**Table 2:** Relations between recurrence patterns and expressions of human gastric mucin, MUC6, MUC2 and CDIO

	**Peritoneal (*n*=60)**	**Haematogenous (*n*=32)**	**Locoregional (*n*=18)**	**Control (*n*=116)**
*Human gastric mucin*^a^				
Negative	16 (26.7%)	18 (56.3%)	6 (33.3%)	53 (45.7%)
Positive	44 (73.3%)	14 (43.8%)	12 (66.7%)	63 (54.3%)
				
*MUC6*				
Negative	23 (38.3%)	18 (56.3%)	6 (33.3%)	43 (37.1%)
Positive	37 (61.7%)	14 (43.8%)	12 (66.7%)	73 (62.9%)
				
*MUC2*^b^				
Negative	42 (70.0%)	20 (62.5%)	9 (50.0%)	45 (38.8%)
Positive	18 (30.0%)	12 (37.5%)	9 (50.0%)	71 (61.2%)
				
*CD10*^c^				
Negative	51 (85.0%)	18 (56.3%)	15 (83.3%)	89 (76.7%)
Positive	9 (15.0%)	14 (43.8%)	3 (16.7%)	27 (23.3%)

a *P*=0.01 (peritoneal *vs* haematogenous) and *P*=0.022 (peritoneal *vs* control).

b *P*=0.0002 (peritoneal *vs* control) and *P*=0.028 (haematogenous *vs* control).

c *P*=0.0054 (peritoneal *vs* haematogenous) and *P*=0.039 (haematogenous *vs* control).

. The expressions of HGM, MUC6, MUC2 and CD10 were observed in 73.3, 61.7, 30.0 and 15.0% of patients in the peritoneum recurrence group, respectively; in 43.8, 43.8, 37.5 and 43.8% of patients in the haematogenous recurrence group, respectively; in 66.7, 66.7, 50.0 and 16.7% of patients in the locoregional recurrence group, respectively; and in 54.3, 62.9, 61.2 and 23.3% of patients in the control group, respectively. Peritoneal recurrence was significantly associated with HGM-positive tumours (*P*=0.022) and MUC2-negative tumours (*P*=0.0002), compared with the control group. Haematogenous recurrence was significantly associated with MUC2-negative tumours (*P*=0.028) and CD10-positive tumours (*P*=0.039), compared with the control group. No significant differences in expression were observed between the locoregional recurrence group and the control group.

### Relations between recurrence patterns and the phenotypic marker expression patterns of the tumours

The relations between the recurrence patterns and the phenotypic marker expression patterns of the tumours are shown in Table 3

**Table 3:** Relations between recurrence patterns and the phenotypic marker expression pattern of the tumour

	**Peritoneal (*n*=60)**	**Haematogenous (*n*=32)**	**Locoregional (*n*=18)**	**Control (*n*=116)**
Gastric phenotype^a^	35 (58.3%)	8 (25.0%)	8 (44.4%)	33 (28.4%)
				
Gastric and intestinal mixed phenotype^b^	16 (26.7%)	12 (37.5%)	8 (44.4%)	60 (51.7%)
				
Intestinal phenotype	5 (8.3%)	8 (25.0%)	2 (11.1%)	18 (15.5%)
				
Unclassified phenotype	4 (6.7%)	4 (12.5%)	0 (0%)	5 (4.3%)

a *P*=0.0046 (peritoneal *vs* haematogenous) and *P*=0.0002 (peritoneal *vs* control).

b *P*=0.0025 (peritoneal *vs* control).

. G-, GI-, I- and UC-phenotype tumours composed 58.3, 26.7, 8.3 and 6.7% of the peritoneum recurrence group, respectively; 25.0, 37.5, 25.0 and 12.5% of the haematogenous recurrence group, respectively; 44.4, 44.4, 11.1 and 0% of the locoregional recurrence group, respectively; and 28.4, 51.7, 15.5 and 4.3% of the control group, respectively. Peritoneal recurrence was significantly associated with G-phenotype tumours, compared with the control group (*P*=0.0002). No significant differences in the phenotypic marker expression patterns of tumours in the haematogenous or locoregional recurrence groups were found, when compared with the control group.

## DISCUSSION

Several studies on the relationship between recurrence patterns of gastric carcinoma after surgery and clinicopathological characteristics have been performed (Maehara *et al*, 1996; Yoo *et al*, 2000; Ohno *et al*, 2003; Roviello *et al*, 2003). Reported risk factors for peritoneal recurrence include an undifferentiated histologic type, infiltrative tumour growth and serosal invasion (Maehara *et al*, 1996; Yoo *et al*, 2000; Roviello *et al*, 2003). Reported risk factors for haematogenous recurrence, on the other hand, include blood vessel invasion, a differentiated histologic type and expansive tumour growth (Maehara *et al*, 1996; Yoo *et al*, 2000; Ohno *et al*, 2003). In the present study, peritoneal recurrence was significantly associated with a larger tumour size, a deeper depth of invasion, and higher incidences of tumours with whole tumour location, undifferentiated-type tumours, infiltrative growth tumours and lymph node metastasis, compared with the control group, while haematogenous recurrence was significantly associated with a higher mean age, a higher male-to-female ratio, and higher incidences of blood vessel invasion and lymph node metastasis. Therefore, clinicopathological analysis appears to be a useful approach predicting recurrence patterns after surgery. Furthermore, our present results clearly show that recurrence patterns after curative resection vary with the gastric and intestinal phenotypic marker expression of the tumour. To the best of our knowledge, this is the first report describing an association between phenotypic marker expression in gastric carcinomas and the recurrence pattern after surgery.

Our present results revealed that the incidences of HGM-positive tumours and MUC2-negative tumours were significantly higher in the peritoneal recurrence group than in the control group. Taking into account the expression combinations of HGM, MUC6, MUC2 and CD10, the incidence of G-phenotype tumours was significantly higher in the peritoneal recurrence group than in the control group. With respect to the relations between the clinicopathological findings and HGM expression, we previously reported that HGM expression was correlated with a higher proportion of infiltrative tumours and undifferentiated-type tumours in advanced gastric carcinomas; we also found a higher incidence rate of infiltrative tumours in G-phenotype tumours than in I-phenotype tumours (Tajima *et al*, 2001b). Even differentiated gastric carcinomas with a G-phenotype have been reported to be more likely to transform into undifferentiated carcinomas and exhibit infiltrative growth to deeper layers of the invaded surrounding structures through the loss of E-cadherin function, compared with differentiated gastric carcinomas with an I-phenotype (Endoh *et al*, 1999; Saito *et al*, 2001; Tajima *et al*, 2001b). Undifferentiated carcinomas with infiltrative growth have been reported to be mainly noted in cases with an advanced level of invasion into the serosal layer or adjacent organs (Maehara *et al*, 1996). As described above, our present results showed that peritoneal recurrence was dominant among undifferentiated tumours and tumours exhibiting serosal invasion, infiltrative tumour growth and lymph node metastasis; these clinicopathological characteristics have also been correlated with G-phenotype and HGM-positive tumours (Tajima *et al*, 2001b). In these cases, the cancerous cells are thought to infiltrate the gastric wall, penetrate the serosa and disseminate trans-serosally to the peritoneum. Therefore, the association between G-phenotype or HGM-positive tumours and peritoneal recurrence in the present study may be attributed to the clinicopathological characteristics of the G-phenotype or HGM-positive tumours.

Haematogenous metastasis is thought to mainly occur when cancerous cells released from the primary site enter the blood vessel system and are transported to a target organ, where attachment and proliferation occurs (Maehara *et al*, 1996). Thus, blood vessel invasion is considered to be one of the most important factors for haematogenous metastasis. In the present study, the incidences of MUC2-negative tumours and CD10-positive tumours were significantly higher in the haematogenous recurrence group than in the control group. Concerning the relations between the clinicopathological findings and MUC2 expression, Utsunomiya *et al* (1998) reported that MUC2 expression in tumours was correlated with lower levels of invasion and lymph node metastasis in gastric carcinomas. In the present study, peritoneal recurrence was also strongly associated with MUC2-negative tumours. Among colorectal carcinomas, MUC2-positive tumours have been reported to have a relatively good prognosis, with a low incidence of liver metastasis (Hanski *et al*, 1997). Therefore, MUC2 expression might be inversely correlated with tumour aggressiveness in gastric carcinomas. As for the relations between the clinicopathological findings and CD10 expression, we previously reported that CD10 expression was correlated with a higher male-to-female ratio of patients, a differentiated histologic type, expansive tumour growth and blood vessel invasion in advanced gastric carcinomas (Tajima *et al*, 2001b); these clinicopathological characteristics have also been associated with haematogenous metastasis (Maehara *et al*, 1996; Yoo *et al*, 2000; Roviello *et al*, 2003). We also reported a higher incidence rate of synchronous liver metastasis in CD10-positive tumours than in CD10-negative tumours (Tajima *et al*, 2001b). Among colorectal carcinomas, CD10-positive tumours exhibited a higher incidence of blood vessel invasion and were at increased risk of liver metastais (Yao *et al*, 2002). Moreover, an ultrastructural study by Shimizu *et al* (2000) revealed that microvilli, as demonstrated by CD10 expression, were generated on the luminal surface of metastatic liver adenocarcinomas. Based on these previous data and the present findings, CD10-positive gastric carcinomas appear to have a strong tendency toward blood vessel invasion, leading to haematogenous metastasis.

In the present study, we demonstrated that the recurrence patterns after curative resection, such as peritoneal and haematogenous recurrences, vary with the phenotypic marker expression of the tumour. Therefore, evaluating the gastric and intestinal phenotypic marker expression of tumours may be useful for predicting the recurrence patterns of the gastric carcinomas after surgery. Careful postoperative follow-up is vital for patients with a high-risk peritoneal or haematogenous recurrence, since the prognosis of patients with recurrence is very poor; additional and intensive therapies after surgery may be indicated for such cases. The results of several randomised trials have demonstrated that intraperitoneal chemotherapy in normothermic or hyperthermic patients tends to improve survival rates and decrease the incidence of peritoneal failure compared with surgery alone (Yu *et al*, 1998; Fujimoto *et al*, 1999). For patients with a high risk of peritoneal recurrence, intraperitoneal chemotherapy may thus be a valuable adjuvant at operation or early in the postoperative period.

Obvious differences in the biological behaviour of tumours with different phenotypic marker expressions have been reported. Kabashima *et al* (2002) reported that G-phenotype tumours could potentially degrade the extracellular matrix through the overexpression of matrix metalloproteinases, compared with I-phenotype tumours. Shibata *et al* (2003) reported that the apoptotic index/proliferative index ratio was significantly lower in G-phenotype tumours than in I-phenotype tumours. We previously reported that patients with G-phenotype tumours have a poorer prognosis than those with I-phenotype tumours among patients with advanced gastric carcinoma (Tajima *et al*, 2001b). We also previously reported that postoperative chemotherapy with 5-FU was effective for patients with G-phenotype tumours, since the incidence of intratumoral expression of thymidylate synthase, the target enzyme of 5-FU, was significantly low in G-phenotype tumours (Tajima *et al*, 2003). These previous data and our present findings suggest that appropriate postoperative follow-up programmes and therapeutic approaches may differ according to the phenotypic marker expression of the tumour.

In conclusion, our present findings show that the gastric and intestinal phenotypic marker expression of the tumour, determined by the HGM, MUC6, MUC2 and CD10 expression patterns, may be used to predict the recurrence pattern of gastric carcinomas after curative resections.
